# Oronasal mucosal melanoma is defined by two transcriptional subtypes in humans and dogs with implications for diagnosis and therapy

**DOI:** 10.1002/path.6377

**Published:** 2025-01-19

**Authors:** Kelly L Bowlt Blacklock, Kevin Donnelly, Yuting Lu, Jorge del Pozo, Laura Glendinning, Gerry Polton, Laura Selmic, Jean‐Benoit Tanis, David Killick, Maciej Parys, Joanna S Morris, Inge Breathnach, Stefano Zago, Sara M Gould, Darren J Shaw, Michael S Tivers, Davide Malucelli, Ana Marques, Katarzyna Purzycka, Matteo Cantatore, Marie E Mathers, Mark Stares, Alison Meynert, E Elizabeth Patton

**Affiliations:** ^1^ Royal (Dick) School of Veterinary Studies and the Roslin Institute Edinburgh UK; ^2^ MRC Human Genetics Unit, Institute of Genetics and Cancer University of Edinburgh Edinburgh UK; ^3^ Edinburgh Cancer Research, CRUK Scotland Centre, Institute of Genetics and Cancer University of Edinburgh Edinburgh UK; ^4^ North Downs Specialist Referrals Bletchingley UK; ^5^ Department of Veterinary Clinical Sciences The Ohio State University Columbus OH USA; ^6^ Department of Small Animal Clinical Sciences, Institute of Infection, Veterinary and Ecological Science University of Liverpool Neston UK; ^7^ University of Glasgow Glasgow UK; ^8^ The Ralph Veterinary Referral Centre Marlow UK; ^9^ University of Bristol Bristol UK; ^10^ Paragon Veterinary Referrals, Paragon Point, Red Hall Crescent Wakefield UK; ^11^ VetsNow Glasgow UK; ^12^ Anderson Moores Veterinary Specialists, The Granary, Bunstead Barns Hampshire UK; ^13^ Department of Pathology Western General Hospital Edinburgh UK; ^14^ Edinburgh Cancer Centre, Western General Hospital, Crewe Road Edinburgh UK

**Keywords:** mucosal melanoma, canine, human, oronasal, transcriptome, CTLA4, MET, immunity, microbiome, macrophages

## Abstract

Mucosal melanoma is a rare melanoma subtype associated with a poor prognosis and limited existing therapeutic interventions, in part due to a lack of actionable targets and translational animal models for preclinical trials. Comprehensive data on this tumour type are scarce, and existing data often overlooks the importance of the anatomical site of origin. We evaluated human and canine oronasal mucosal melanoma (OMM) to determine whether the common canine disease could inform the rare human equivalent. Using a human and canine primary OMM cohort of treatment‐naive archival tissue, alongside clinicopathological data, we obtained transcriptomic, immunohistochemical, and microbiome data from both species. We defined the transcriptomic landscape in both species and linked our findings to immunohistochemical, microbiome, and clinical data. Human and dog OMM stratified into two distinctive transcriptional groups, which we defined using a species‐independent 41‐gene signature. These two subgroups are termed CTLA4‐high and MET‐high and indicate actionable targets for OMM patients. To guide clinical decision‐making, we developed immunohistochemical diagnostic tools that distinguish between transcriptomic subgroups. We found that OMM had conserved transcriptomic subtypes and biological similarity between human and canine OMM, with significant implications for patient classification, treatment, and clinical trial design. © 2025 The Author(s). *The Journal of Pathology* published by John Wiley & Sons Ltd on behalf of The Pathological Society of Great Britain and Ireland.

## Introduction

Mucosal melanoma (MM) is a rare and aggressive form of melanoma arising from melanocytes in sun‐protected mucosal surfaces, including oral, nasal, genital, and anorectal regions [1]. Despite accounting for a minority (1.4%) of melanoma cases, MM carries a disproportionately higher morbidity and mortality risk than cutaneous melanoma (CM) [2]. MM is often diagnosed at an advanced stage, exhibits a higher likelihood of metastasis [3, 4], and a reduced 5‐year survival [3, 5, 6, 7]. Unlike CM, MM lacks well‐defined precursor lesions and has few oncogenic driver mutations or effective therapeutics [2, 3, 8, 9, 10]. MM from different anatomical sites is mutationally heterogeneous, although interestingly, there is some evidence for ‘upper’ and ‘lower’ body site‐specific mutational profiles [4].

We reasoned that exploring the nuances of anatomical site‐specific MM, with a specific focus on oronasal mucosal melanoma (OMM), had the potential to disentangle the inherent heterogeneity of this rare melanoma subtype. There are few experimental models for OMM; however, OMM is a common and naturally occurring oral tumour in dogs [11, 12, 13, 14]. Previous research identified two molecular subgroups in OMM in dogs based on RNA expression, but the clinical significance of these subgroups for veterinary oncology is unknown [15]. Despite dogs being proposed as a model for human OMM, it is unknown whether these subtypes even exist in human OMM and whether human OMM is analogous to the canine disease. These outstanding questions are critical because the lack of diagnostic subtypes precludes subtype classification that could be pivotal for patient‐centric precision medicine and could inform clinical trial design to account for potential subtype‐specific responses [9, 16, 17, 18, 19, 20, 21].

Here, for the first time, we present transcriptomic and 16S sequencing data side by side for two new cohorts of human and canine OMM. Our analysis reveals OMM tumour stratification for both human and canine patients based on a CTLA4‐high or MET‐high transcriptional signature, defined by 41 genes, shared between species. This patient stratification is facilitated by an immunohistochemical tool, pivotal not only for individualised patient care but also for informing clinical trial design.

## Materials and methods

### Ethics statement and sample collection

This study was conducted with the approval of the University of Edinburgh's Veterinary Ethical Review Committee (Reference no. 10.21) and Lothian BioResource Access Committee (REC reference 20/ES/0061 SR1725).

Formalin‐fixed, paraffin‐embedded (FFPE) human and canine OMM tissue samples and associated clinical data were collected from patients with naturally occurring tumours who had undergone surgical resection or biopsy of the primary tumour. Tissue was only included in the study with the informed, written consent of the human patient or the caregiver of the dog who bore the tumour. The treatment that a human or canine patient received was unaffected by their inclusion in this study.

FFPE tissue samples of treatment‐naïve human OMMs were collected between 2006 and 2021. Clinicopathological data collected included patient gender, ethnicity, age (years), primary tumour location, presence of ulceration, tumour depth (mm), World Health Organization (WHO) stage, metastatic status, provision of adjuvant therapy, and duration of patient survival or lost to follow‐up (in months following initial diagnosis of OMM).

FFPE tissue samples of treatment‐naïve canine OMMs were collected between 2011 and 2021 for histopathology from dogs attending the Clinical Veterinary Oncology departments at the Universities of Edinburgh, Bristol, Liverpool, Glasgow and Ohio Small Animal Teaching Hospitals, Vets Now (Glasgow) Referrals, Paragon Referrals, The Ralph Veterinary Referral Centre, Anderson Moores Veterinary Specialists, and North Downs Specialist Referrals. Clinicopathological data collected included sex, breed, age (years), primary tumour anatomical location, melanoma type (melanotic/amelanotic, pleomorphic/composite/nevocytoid/animal, ulcerated, exophytic), depth of invasion (mm), stroma:tumour ratio (%), mitotic count (%), metastatic status (confirmed by abdominal ultrasound, thoracic computed tomography or radiography, and/or histological examination of ≥1 regional lymph node), WHO stage, provision of adjuvant therapy, and duration of patient survival or lost to follow‐up (in months following initial diagnosis of OMM).

The diagnosis of OMM was confirmed by a consultant histopathologist (MM) or board‐certified veterinary anatomic pathologist (JdP), and the regions of the FFPE blocks that represented OMM tissue tumour identified.

### RNA and DNA extraction

Using a manual tissue arrayer (Beecher MTA‐1, Estigen OÜ, Tartu, Estonia) machine, two cores (1 mm outer diameter, 5 mm length) were harvested from each canine FFPE block: one from an area of OMM and one from histopathologically normal tissue. RNA and DNA were isolated from the tissue cores using a Covaris E220 Evolution Focused Ultrasonicator (Covaris Ltd, Woddington, Brighton, UK, Catalogue No. 500429) and the truXTRAC® FFPE total NA (Nucleic Acid) Kit – Column (Covaris, Catalogue No. 520220). Inhibitors (e.g. melanin) were removed using the OneStep PCR Inhibitor Removal kit (Zymo Research, Irvine, CA, USA, Catalogue No. D6030).

#### Quality control

DNA (canine only) and total RNA (human and canine) were assessed for quality and integrity using a Fragment Analyser Automated Capillary Electrophoresis System (Agilent Technologies, Santa Clara, CA, USA, Catalogue No. 5300) with the Genomic DNA 50 kb Kit (Agilent Technologies, Catalogue No. DNF‐467‐0500) and Standard Sensitivity RNA Analysis Kit (Agilent Technologies, Catalogue No. DNF‐471‐0500) respectively, and then assessed using a Qubit 2.0 Fluorometer (Thermo Fisher Scientific, Waltham, MA, USA, Catalogue No. Q32866) and the Qubit DNA (Thermo Fisher Scientific, Catalogue No. Q32853) or RNA (Thermo Fisher Scientific, Catalogue No. Q10210) broad range assay kits. In the RNA samples, DNA contamination was quantified using a Qubit dsDNA HS assay kit (Thermo Fisher Scientific, Catalogue No. Q32854). The Fragment Analyser data showed a high degree of RNA degradation, so the fragmentation step was omitted from the library preparation protocol.

#### RNA library preparation

First‐strand cDNA was generated from 50 ng of each total RNA sample using a SMARTer® Stranded Total RNA‐Seq Kit version 2 – Pico Input Mammalian kit (Clontech Laboratories, Mountain View, CA, USA, Catalogue No. 634411). Illumina‐compatible adapters and indexes were then added via five cycles of PCR. AMPure XP beads (Beckman Coulter, Brea, CA, USA, Catalogue No. A63881) were then used to purify the cDNA library. Depletion of ribosomal cDNA (cDNA fragments originating from highly abundant rRNA molecules) was performed using ZapR version 2 and R‐probes version 2 specific to mammalian ribosomal RNA and human mitochondrial rRNA. Uncleaved fragments were then enriched by 13 cycles of PCR before a final library purification using AMPure XP beads. Libraries were quantified with the Qubit dsDNA HS assay and assessed for quality and size distribution of library fragments using the Fragment Analyser and a NGS Fragment Kit (Agilent Technologies, Catalogue No. DNF‐473‐0500).

#### DNA library preparation

Libraries were prepared from up to 300 ng of each DNA sample using the Sure Select XT Target Enrichment for Illumina System according to the provided protocol for FFPE samples. DNA samples were sheared to an average fragment size of 150–200 bp using a Covaris E220 Evolution and purified using Agencourt AMPure XP beads (Beckman Coulter, Catalogue No. A6881). Purified DNA fragments were end‐repaired to remove 3’‐ and 5’ overhangs before further purification with AMPure XP beads. A single ‘A' nucleotide was added to the 3’ ends of the blunt fragments to prevent them from ligating to another during the subsequent adapter ligation reaction, and a corresponding single ‘T' nucleotide on the 3’ end of the adapter provided a complementary overhang for ligating the adapter to the fragment. dA‐tailed DNA was again purified using AMPure XP beads before paired‐end adapters were ligated to the ends of the dA‐tailed DNA fragments to prepare them for hybridisation in a flow cell. After purification using AMPure XP beads, 13 cycles of PCR were used to selectively enrich those DNA fragments that had adapter molecules on both ends and amplify the amount of DNA in the library suitable for target enrichment. Amplified gDNA libraries were purified using AMPure XP beads. gDNA libraries were quantified by fluorometry using a Qubit dsDNA HS assay and assessed for quality and fragment size using the Agilent bioanalyser using a DNA HS Kit (Agilent Technologies, Catalogue No. 5067‐4626). Seven hundred fifty ng of each gDNA library (where available) was hybridised to target‐specific probes (Canine All‐Exon) before target molecules were captured on streptavidin beads and non‐target molecules removed with a series of washes. Individual hybridised and captured libraries were then amplified using 12 cycles of PCR (with unique indexing primers to allow multiplexed sequencing) and purified with AMPure XP beads.

Indexed libraries were measured by fluorometry using a Qubit dsDNA HS assay and assessed for quality and fragment size using the Agilent bioanalyser with a DNA HS Kit. DNA concentration and fragment size data were used to calculate molarity for sequencing.

#### Sequencing

Sequencing was performed using the NextSeq 2000 platform (Illumina, San Diego, CA, USA, Catalogue No. 20038897) with NextSeq 2000 P3 Reagents (200 Cycles) (Illumina, Catalogue No. 20040560). Libraries were combined in equimolar pools based on Qubit and bioanalyser assay results, and each pool was sequenced in a P3 flow cell. For RNA sequencing (RNA‐seq), PhiX Control version 3 (Illumina, Catalogue No. FC‐110‐3001) was spiked into each run at a concentration of 1% to allow troubleshooting in the event of any issues. Base call data produced by the NextSeq 1000/2000 Control Software (version 1.4.1.39716) were automatically converted into FASTQ files and uploaded to BaseSpace.

### Immunohistochemistry

#### Human OMM

Immunohistochemistry was conducted on 3‐μm‐thick FFPE human OMM tissue using a Leica Bond immunostainer (Leica Bond III Autostainer, Leica, Sheffield, UK, Catalogue No. 21.2201). A standard protocol was followed in accordance with the manufacturer's instructions. Anti‐CD3 was used to identify T cells and natural killer (NK) cells (1:500 Novocastra Liquid Mouse Monoclonal Antibody CD3, Leica Biosystems, Sheffield, UK, Catalogue No. NCL‐L‐CD3‐565), Anti‐CD19 was employed to stain B‐lymphocytes (1:50 Novocastra Liquid Mouse Monoclonal Antibody CD19, Leica Biosystems, Catalogue No. NCL‐L‐CD19‐163), Anti‐CD68 was used to label monocytes and macrophages (1:50 Monoclonal Mouse Anti‐Human CD68, Dako, Santa Clara, CA, USA, Catalogue No. M0876). Tissue was also stained for CD163 (1:50, CD163 Monoclonal Antibody, Thermo Fisher Scientific, Catalogue No. MA5‐11458), CTLA4 (1:50 CTLA4 Polyclonal Antibody, Invitrogen, Darmstadt, Germany, Catalogue No. PA5‐115060), and MET (1:400 HGFR/c‐MET Antibody, 1G7NB, Novus Biologicals, Abingdon, Oxford, UK, Catalogue No. 44306SS) using the same protocol.

#### Canine OMM

Immunohistochemistry was conducted on 4‐μm‐thick FFPE canine OMM tissue, cut using a microtome (Thermo Fisher Scientific Rotary Microtome Microm HM 340 E). All tissue sections, including relevant controls, were deparaffinised in xylene and rehydrated in graded ethanol through to distilled H_2_O prior to staining. Heat‐induced antigen retrieval was performed using sodium citrate (pH 6.0; 110 °C; 5–12 min overall) in a microwave (HistoS5, Milestone, Sorisole, Italy). Once retrieved, all tissue sections were stained in relevant batches in an autostainer (Autostainer 360, Epredia, Runcorn, UK) following a standard protocol that incorporates a 30‐min incubation with the primary antibody at room temperature. Anti‐CD3 was used to identify T cells and NK cells (1:200 Novocastra Liquid Mouse Monoclonal Antibody CD3, Leica Biosystems, Catalogue No. NCL‐L‐CD3‐565), anti‐PAX5 was employed to identify B lymphocytes (1:50 Mouse Monoclonal Antibody Pax5, Becton and Dickinson, Workingham, UK, Catalogue No. P67320‐050), and anti‐IBA1 was used to label monocytes and macrophages (1:500 Rabbit polyclonal anti‐Iba1, FUJIFILM Wako, Richmond, VA, USA, Catalogue No. 019‐19741). All sections were treated with Dako Real Peroxidase Blocking solution (Dako S2023) to block endogenous peroxidases. The next step was a 40‐min incubation with either EnVision anti‐Mouse (Dako, EnVision+ System HRP‐labelled polymer; Catalogue No. K400111‐2) or EnVision anti‐Rabbit (Dako, EnVision+ System HRP‐labelled polymer; K400311‐2). This system is based on an HRP‐labelled polymer conjugated with secondary antibodies. The labelled polymer does not contain avidin or biotin. As such, nonspecific staining resulting from endogenous avidin‐biotin activity is eliminated or significantly reduced. Envision mouse was used to stain tissues for CD3 and PAX5, whereas EnVision Anti‐Rabbit with DAB enhancer protocol was used for IBA1 [Dako EnVision® + Dual Link System‐HRP (DAB+), Catalogue No. PD04048_02/K4065]. All sections were then counterstained with Harris' haematoxylin (Varistain Gemini Slide Stainer, Thermo Fisher Scientific), dehydrated with graded ethanol through to xylene, and coverslipped. Canine tissue was stained for CTLA4 and MET using the same immunohistochemical protocol as for human OMM.

### Image acquisition

Slides were digitally scanned (NanoZoomer‐XR, Hamamatsu Photonics K.K, Shizuoka, Japan) and raw image data were saved in ndpi format and handled by the software ndp.view 2 (Hamamatsu Photonics) to save details of the whole image in JPEG format. Images were imported to ImageJ (FiJi Project, 2.11.0, GPLv3+) for labelling and uploaded to QuPath (version 0.5.0) [22].

### 16S sequencing

DNA was isolated, purified, and quantified from FFPE core tissue samples from human and canine OMM as described earlier. DNA was similarly isolated, purified, and quantified from four FFPE core samples of normal canine oral tissue. Libraries were prepared from 2 μl of each purified DNA sample using a Quick‐16S™ Plus NGS Library Prep Kit (V3‐V4, UDI) (Zymo Research, Catalogue Nos. D6421‐PS1 and D6421‐PS2) following the manufacturer's protocol. Alongside the experimental samples, control libraries were prepared from the ZymoBIOMICS™ Microbial Community DNA Standard (positive), a ‘blank’ sample taken through the DNA extraction and purification processes without tissue (negative) and nuclease‐free water (nontemplate control). The source material for both the positive control and NTC were provided in the Quick‐16S™ Plus kit. The pool was purified using AMPure XP beads (Beckman Coulter, Catalogue No. A63881) to remove primer‐dimer sequences, and DNA concentration was assessed using fluorometry (Qubit dsDNA High Sensitivity Assay, Thermo Fisher Scientific, Catalogue No. Q32851) and assessed for quality and fragment size using an Agilent bioanalyser with the DNA High Sensitivity Kit (Agilent, Catalogue No. 5067‐4626). Fragment size and quantity measurements were used to calculate molarity for sequencing.

Sequencing was performed using the NextSeq 2000 platform (Illumina, Catalogue No. SY‐415‐1002) using NextSeq 1000/2000 P1 Reagents (600 cycles) version 3 Kit (Catalogue No. 20075294). Loading concentration was 750 pm as recommended in the Quick‐16S Plus Kit User Guide. PhiX Control version 3 (Catalogue No. FC‐110‐3001) library was spiked in at 40% to help with cluster resolution and facilitate troubleshooting in case of any problems with the run.

### Expression data analysis pipeline

#### Alignment and gene‐level counts

RNA‐seq data were processed using the nf‐core ‘rnaseq’ pipeline version 3.8.1 [23, 24, 25, 26, 27, 28, 29, 30, 31, 32, 33, 34, 35, 36, 37]. In brief, samples were aligned using STAR version 2.6.1d and gene‐based counts produced using Salmon version 1.5.2. Canine data were aligned to the ROS_Cfam_1.0 reference genome, annotated using the corresponding GTF file for build accession GCA_014441545.1. Human sequence data were aligned to hg38 and annotated with GENCODE version 41 [38].

#### Unsupervised clustering

Unsupervised consensus clustering of expression data was performed using the R/Bioconductor package cola [39]. Prior to clustering, gene‐level count data were subjected to a variance‐stabilising transformation using DESeq2 [34], and the resulting matrix was used as input. Five different clustering algorithms were tested, with an evaluation of two to six clusters in each case. The cola algorithm resamples count data a fixed number of times, repeating the clustering process on each iteration. Upon completion, cola may present multiple stable solutions, with partitions varying in size and number. When determining which of these outputs to investigate, the best solution was selected based on the highest performance measured by the silhouette score, concordance, and proportion of ambiguous clustering (PAC) score.

#### Differential expression analysis

All downstream differential expression analysis was performed using DESeq2. Models were fitted treating the clusters identified by cola, sex, and, where appropriate, batch as factors. Log fold‐change (logFC) estimates were produced using the apeglm shrinkage method [40], which is intended to provide more robust estimates in the event of high within‐group variability. Shrunken logFC estimates were accompanied by *s*‐values [41], an aggregate false sign rate, which are broadly analogous to *q* values. *p* values were also computed and adjusted using the independent hypothesis weighting (IHW) method [42]. Results were annotated using biomaRt [43], and volcano plots were generated using EnhancedVolcano [44].

#### Over‐representation analysis

Over‐representation analysis (ORA) was performed using the R package ClusterProfiler [45]. ORA was performed for up‐ and downregulated genes independently, taking the top differentially expressed genes with an *s*‐value <0.01 in each case. These gene lists were then tested for over‐representation against the Biological Process (BP), Cellular Component (CC), and Molecular Function GO ontologies [46], in addition to known pathways from the Kyoto Encyclopedia of Genes and Genomes (KEGG) [47]. Minimum and maximum gene set sizes were set to 10 and 1,000 respectively for all analyses.

### Immunocyte deconvolution

The computational framework CIBERSORTx was used to deconvolute the tumour‐infiltrating immune cells from bulk RNA‐sequencing data [48]. Twenty‐two immune cell subtypes were parsed from the annotated gene signature matrix LM22 and 100 permutations of the CIBERSORTx web portal [48]. After running, only samples with CIBERSORT *p* values <0.05 were included in subsequent analyses. B‐mode batch correction was applied, and quantile normalisation was disabled to generate absolute scores. CIBERSORT fractions for naïve B cells, memory B cells, and plasma cells were aggregated into B cells; monocytes, M0, M1, M2 macrophages into ‘monocytes and macrophages’; and CD8, CD4, T follicular helper cells, regulatory T cells, and gamma delta T cells into T cells; and resting and activated NK cells into NK cells. Wilcoxon tests were used to compare the immune cell fractions between species, metastatic status, and transcriptomic consensus clustering groups. A Cox regression model was used to describe the time‐to‐event outcomes, with the adjustedCurves package [49] used to show the effect of a continuous variable adjusted for age and gender/sex. Receiver operating characteristic (ROC) curves were plotted, and optimal cut‐off points were defined using Youden's index. Statistical significance was set at *p* < 0.05.

### Image data analysis

Using the HE slides to identify the tumour margins, the tumour areas were defined in QuPath [22]. Positive cell detection was performed using the default parameters, except for sigma, which was set at 1.0 μm. Wilcoxon tests were used to compare the ‘number of positive cells detected per mm^2^’ between transcriptomic consensus clustering groups. Optimal cut‐off points were defined as above. Statistical significance was set at *p* < 0.05.

### 16S analysis

Cutadapt (version 4.5) was used to remove primers from paired‐end *16S* rRNA gene reads [50]. Both paired‐end reads were removed from further analyses if one or both of the pairs did not contain the 16S primers using the –discard‐untrimmed option. Mothur (version 1.48.0) was used for quality control, taxonomic assignment, and operational taxonomic unit (OTU) clustering [51], following an adjusted version of the mothur Miseq pipeline [52]. Sequences were removed if they were >626 bp in length, <322 bp in length, contained ambiguous bases, had homopolymers of >9 bp in length, did not align to the correct variable region of the *16S* rRNA gene, or did not originate from bacteria. Chimeras were identified via the ‘vsearch’ command, then removed. Sequence alignment and taxonomic assignment were conducted using the SILVA database [53] (version 138.1) formatted for use in mothur. OTUs were clustered using the ‘cluster.split’ command in mothur. The OTU file, phylogenetic tree file, taxonomy file, and sample metadata file were packaged into a phyloseq object [54].

Data were decontaminated using decontam (version 1.22.0) [55], whereby contaminants were identified by comparing the prevalence (presence/absence across samples) of each sequence feature in the true positive samples with the prevalence in negative controls. The threshold of 0.5 was established to designate as contaminants exhibiting a higher prevalence in negative controls than positive controls. All contaminant sequence features were then removed from the entire dataset. Thirty‐four contaminants were removed from the dataset, resulting in 33,929 OTUs.

Samples with <3,000 reads were discarded. Two human and one canine sample did not have >3,000 reads and were also discarded, resulting in 11 human OMM samples, 35 canine OMM samples, and four canine normal oral tissue samples.

To adjust for sequencing depth across samples, raw counts for each OTU were converted into relative abundance values by dividing by the total amount of counts for each sample. Goods coverage was calculated using phyloseq‐coverage [54], and samples with a coverage value below 0.9 were considered to have insufficient sequencing depth and were discarded.

To explore the diversity of microbial community composition within samples, we generated alpha‐diversity indices using both Shannon diversity and Chao1 richness. Differences in alpha diversity between cohorts were assessed by applying two‐sided Wilcoxon's rank‐sum tests. *p* values obtained from pairwise correlations were adjusted using the Benjamini–Hochberg procedure to control the false discovery rate. We presented significant findings as box‐and‐whisker plots.

To evaluate whether samples clustered by group according to their overall microbiota compositions, we conducted a beta‐diversity analysis using permutational multivariate analysis of variance (PERMANOVA). This analysis was conducted with the ‘adonis2’ function and Bray–Curtis dissimilarity matrix, implemented in the vegan package in R (version 2.6‐4) [56]. We presented significant findings as principal component analysis (PCA) plots. Statistical significance was defined as *p* < 0.05.

To determine the OTUs most likely to explain differences between groups, we conducted linear discriminant analysis effect size (LefSE) analyses on variables exhibiting statistically significant alpha or beta diversity results. We used the R package microbiomeMarker (1.8.0) [57] to identify markers generated using all phylogenetic levels and presented results as tables and histographs.

## Results

### OMM in humans and dogs share clinicopathological characteristics and poor prognosis

**Figure 1:**
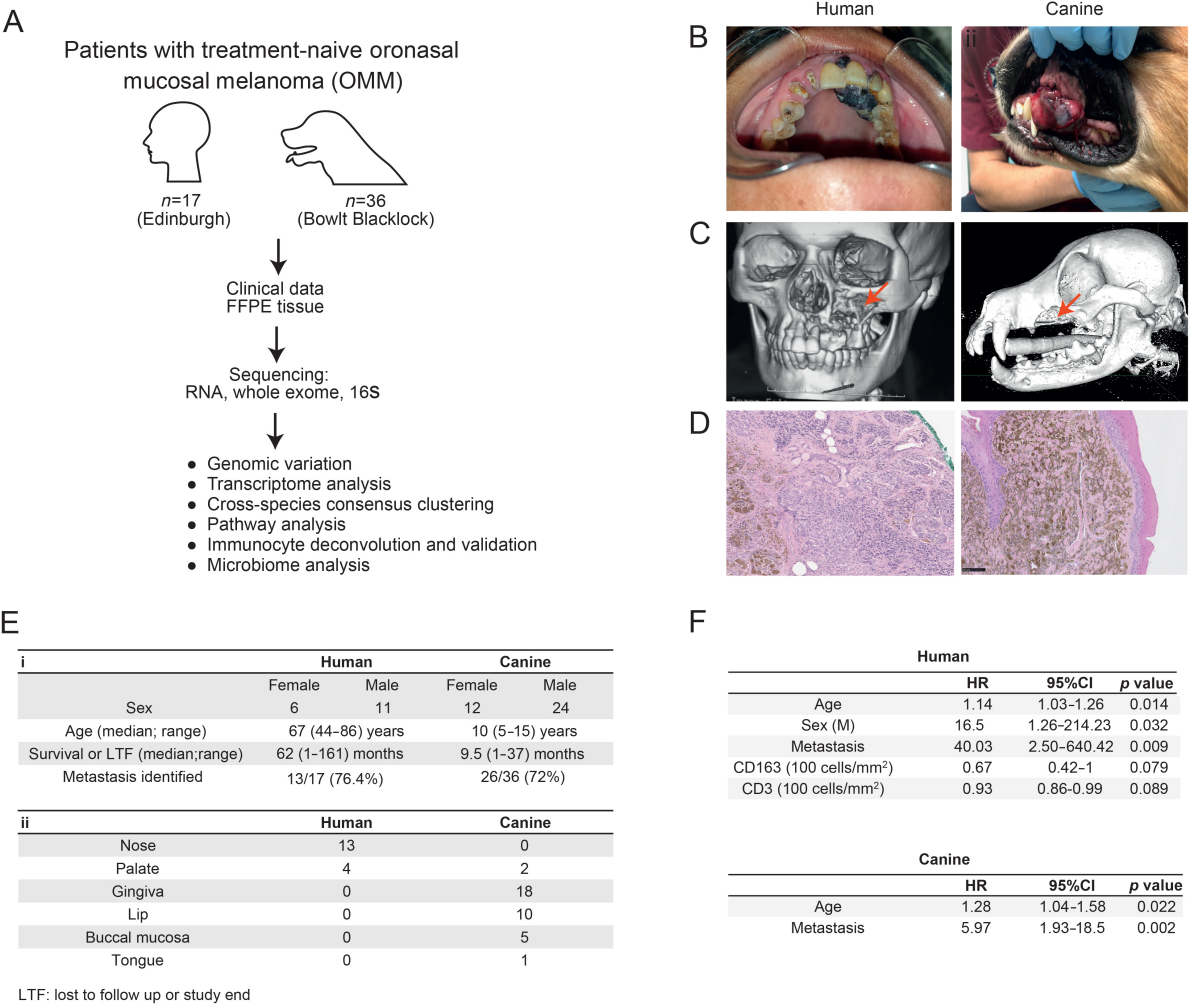
OMM in humans and dogs share clinicopathological characteristics and poor prognosis. (A) Project workflow. (B) Gross appearance of human and canine primary OMM. (C) 3D computed tomography (CT) reconstruction images showing left‐sided maxillary destruction (red arrow) underlying the primary tumour in a human and canine patient. (D) Haematoxylin and eosin staining showing that both human and canine OMM exhibits similar proliferations of pigmented neoplastic infiltrates. (E) Overview of clinical pathological data for the study cohort, encompassing (i) patient data and (ii) anatomical location of the primary OMM. (F) Relevant hazard ratios for survival in human and canine patients with OMM based on clinical parameters and intra‐tumoural immunocyte infiltration. The images of human patients (A and B) were kindly provided by Professor Ankita Chugh BDS MDS, All India Institute of Medical Sciences Jodhpur (copyright retained).

We identified human (Edinburgh cohort; *n* = 17) and canine (Bowlt Blacklock cohort; *n* = 36) patients who presented for treatment of naturally occurring, treatment‐naive OMM and had archival FFPE tissue and clinical records available for analysis (Figure 1A). In both humans and dogs, OMM can appear as melanotic or amelanotic and is most frequently observed in the oronasal cavity in humans and in the oral cavity in dogs (Figure 1B). In both species, underlying bone lysis secondary to local tumour invasion is common (Figure 1C). Histopathological findings are also similar, consisting of proliferations of melanotic or amelanotic infiltrates of pleomorphic, round, and/or spindloid neoplastic cells that invade both the lamina propria and deep connective tissue (Figure 1D). RNA‐seq and 16S sequencing data were compared to clinical data. When considering survival, the time from OMM diagnosis to end of life is not directly comparable between species. Human patients die of their disease, whereas in dogs, 89.3% of recorded deaths are a result of euthanasia [58, 59].

We investigated the relationships between clinicopathological features, treatment, and survival. Clinicopathological data are presented in Figure 1E. The best model to identify predictors of survival in humans included age, gender (male), presence of metastasis, and immunocyte infiltration (Figure 1F). Risk of death increased by 14% for every 1‐year increase in age, 16.5‐fold for male patients, and 40‐fold for patients with metastatic disease (Figure 1F). In dogs, the best model to identify predictors of survival included only age and presence of metastasis (Figure 1F). Human patients who had undergone only biopsy of their OMM without surgery had a significantly increased risk of death compared to those who received curative‐intent surgery (supplementary material, Figure S1A). We found no significant correlation between the relative hazard of death and provision of chemotherapy, radiation therapy, or immunotherapy in humans or dogs (supplementary material, Figure S1A). In our canine patients, reduced survival probability was identified in patients with WHO Stage 3 or 4 disease and ulcerated tumours (supplementary material, Figure S1B,C). Exophytic status, melanotic status, and melanoma histopathological subtype were not associated with survival in dogs (supplementary material, Figure S1D–F). Equivalent data were not available for human patients.

In conclusion, for both human and canine patients with OMM, our clinical data reveal that prognosis is poor and metastasis common. In humans, our data show that curative surgery and being female are associated with improved survival, while radiation, immunotherapy, and chemotherapy do not significantly impact survival in either species.

### OMM can be stratified into two transcriptomic subgroups

**Figure 2:**
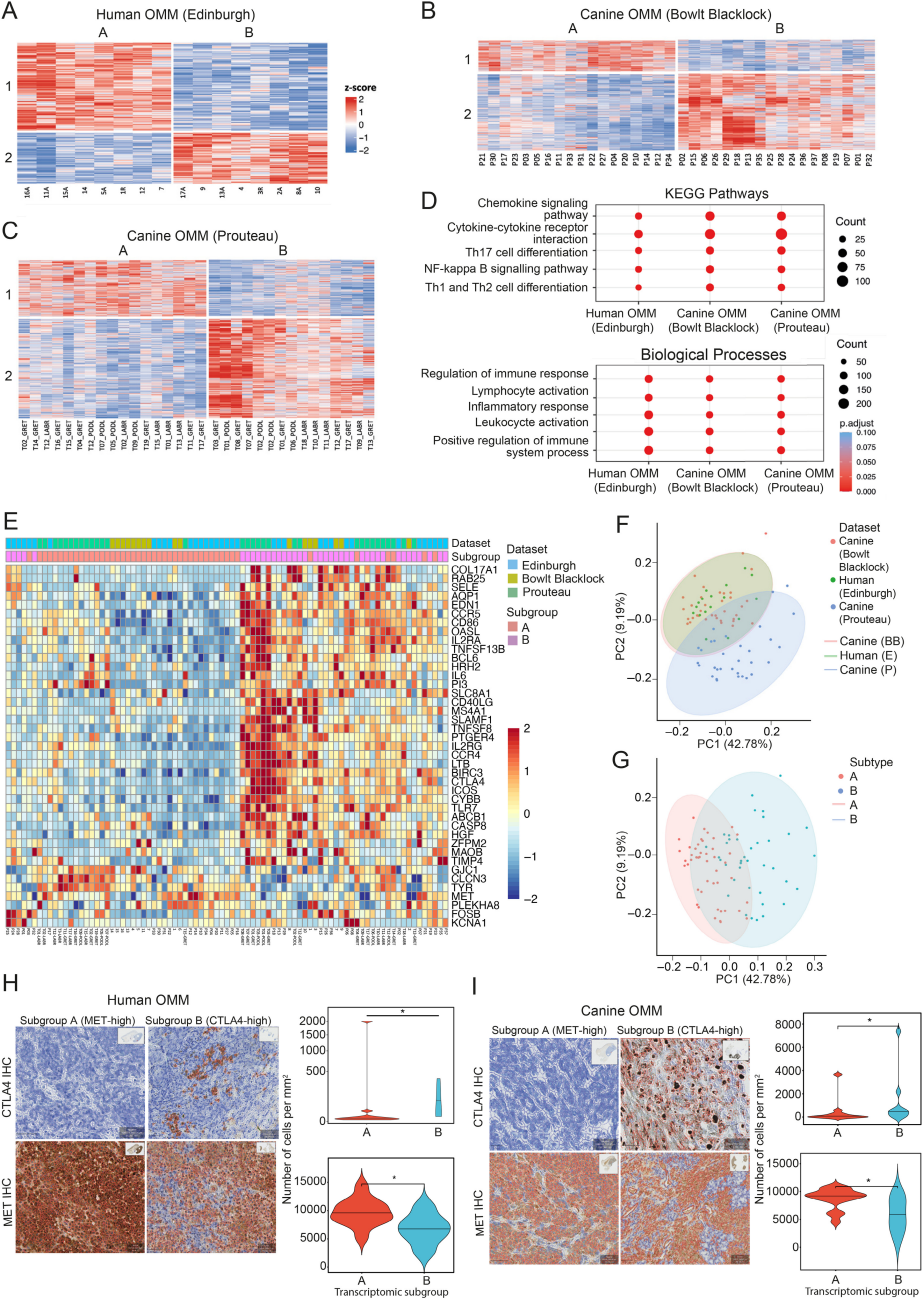
OMM can be stratified into two transcriptomic subgroups. (A–C) Heatmaps of ‘signature genes’, those deemed to differ significantly between groups by cola (F‐test, adj. *p* < 0.05) on bulk RNA‐seq data obtained from OMM from human (Edinburgh; *n* = 17 patients), canine (Bowlt Blacklock; *n* = 36 patients), and canine (Prouteau; *n* = 32 patients) patients. Patients are orientated along the vertical axis, and genes are orientated along the horizontal axis. One human sample was excluded from the plot due to ambiguous group membership (silhouette score <0.5) but was allocated to the most probable group for the purpose of downstream analysis. (D) Transcriptomic subgroup aligned with the immune response in both KEGG pathways and biological processes. (E) Heatmap produced using a 41‐gene signature to stratify patients into transcriptomic subgroups, independent of species. (F and G) PCA plot of 812 homologous genes, stratified according to (F) species and (G) transcriptomic subgroup. (H and I) Violin plots showing MET and CTLA4 IHC markers in (H) human and (I) canine OMM according to transcriptomic subgroup.

Despite no widespread actionable mutation targets in OMM, we hypothesised that OMM might have transcriptional subtypes, as found in CM [60]. We conducted RNA‐seq transcriptomic analysis of OMM from human (Edinburgh) and canine (Bowlt Blacklock) patients and performed unsupervised hierarchical clustering from these cohorts as well as from a published canine OMM RNA dataset [15]. Critically, we identified two stable consensus clusters (A and B) in both species using optimal consensus partitioning with median absolute deviation and spherical k‐means clustering (Figure 2A–C; see Materials and methods). PCA showed minimal overlap between these transcriptional subgroups in all three datasets (supplementary material, Figure S2A). To understand the pathways that characterised the transcriptional subtypes, we applied ORA and showed that KEGG pathways and biological processes aligned with an immune response in consensus cluster group B (Figure 2D). In contrast, ORA of consensus cluster group A was less clear but highlighted pathways associated with the cell cycle and DNA/RNA processing and repair (supplementary material, Data S1).

To investigate whether the transcriptional subgroups shared between human and canine OMM cohorts were mediated through overlapping gene networks, we aimed to find the most important variables that drove the stratification across species and cohorts. We used the machine learning algorithm randomForest [61] and 812 human and canine homologous genes (supplementary material, Data S2). Across five randomly selected seeds, this approach generated successful models (trained on 50% of the samples) that predicted the B subgroup versus the A subgroup in the test data (the remaining 50% of the samples), independently of species (accuracy 79–88%, binomial test, *p* < 0.001). Within these models, 41 genes were consistently ranked among the top 100 genes shared by at least four models, and 17 genes were shared by all five models (supplementary material, Figure S2B and Data S2). Using these 41 genes, optimal discrimination between transcriptomic subgroups was achieved by Euclidean distance clustering (Figure 2E). Using PCA clustering based on these 41 genes, we found that our human OMM and dog OMM RNA‐seq data closely clustered on PCA, indicating strong similarity (Figure 2F). The Prouteau dataset demonstrated a mildly distinct distribution on PC2 (9.19% variation) but not PC1, with the latter strikingly driven by the transcriptional subgroup variation (42.78%) (Figure 2G). Therefore, we concluded that the PC2 difference was mainly caused by technical variations during cohort establishment (Edinburgh and Bowlt Blacklock versus Prouteau), while biologically the two OMM transcriptional subtypes did not exhibit any species‐dependent variations.

Notably, our signature highlighted CTLA‐4 gene expression in both human and dog OMM in subgroup B. This finding was validated by immunohistochemistry (IHC), showing a significantly greater number of CTLA4‐positive cells in the B transcriptomic subgroup (human: *p* = 0.01; canine: *p* = 0.03) (Figure 2H). We therefore termed this subgroup ‘CTLA4‐high’. Of particular interest in subgroup A is a small, enriched panel of genes (*GJC1, CLCN3, TYR, MET, PLEKHAB, FOSB, KCNA1*) highlighted in the 41‐gene signature. Using g:Profiler, we mapped these genes to functional information sources to detect statistically significantly enriched terms, which included the hepatocyte growth factor receptor (HGFR, also known as MET) activity pathway and the MET complex. We performed MET IHC and found MET protein levels to be significantly increased in subgroup A in both humans and dogs (human: *p* = 0.04; canine: *p* = 0.02) (Figure 2I). Therefore, we termed the A subgroup ‘MET‐high’. Additionally, we noticed that the enriched panel of genes were microphthalmia‐associated transcription factor (MITF) target genes and suggest the gene expression signatures of this subgroup may be mediated by MITF [62] (supplementary material, Figure S2C).

In conclusion, we show that human and dog OMM can be stratified into two distinctive transcriptional groups, termed CTLA4‐high and MET‐high, using the same species‐independent 41‐gene signature, providing actionable targets and highlighting the biological similarity of this disease between the two species.

### CTLA4 and macrophages are diagnostic biomarkers for OMM transcriptomic subtypes

The presence of potential drug targets CTLA4 and MET within the subtypes indicated that the stratification of patients into each subtype could inform therapeutic decision‐making and clinical trial design. Because transcriptomic analysis is currently not practical in most clinical settings, we attempted to identify diagnostic IHC markers to discriminate between subtypes.

We reasoned that the CTLA4‐high subtype could be identified by immunocyte infiltrate populations and utilised the computational framework CIBERSORTx to discern the composition of tumour‐infiltrating immune cells from bulk RNA‐seq data [48] (supplementary material, Data S3). In both humans and dogs, total immune cell infiltration (the ‘Absolute Score’) was significantly larger in the CTLA4‐high transcriptomic subgroup (*p* < 0.01 in both species), as well as the total B cells, monocyte/macrophages, and T cells (supplementary material, Figure S3A–C).

**Figure 3:**
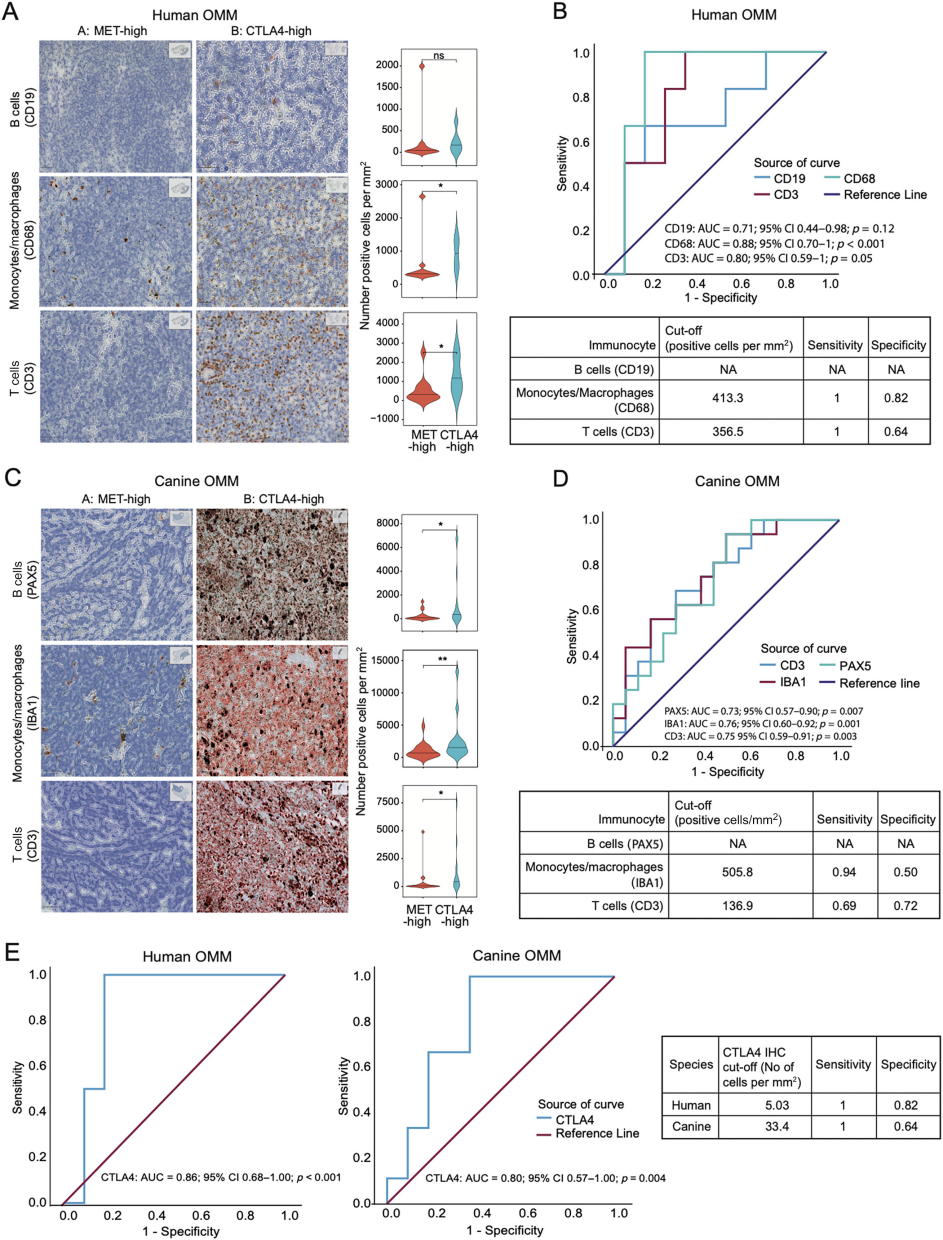
CTLA4 and macrophages are diagnostic biomarkers for the OMM transcriptomic subtypes. (A) Violin plots and representative IHC panels for human OMM, stratified according to transcriptomic subgroup. (B) ROC curves were plotted for each immunocyte IHC marker in (A), and optimal cut‐off parameters were defined. (C) Violin plots and representative IHC panels for canine OMM, stratified according to transcriptomic subgroup. (D) ROC curves were plotted for each immunocyte IHC marker in (C) and optimal cut‐off parameters were defined. (E) ROC curves and optimal cut‐off parameters for CTLA4 in human and canine OMM. *p < 0.05; **<0.01 ns: not significant.

Next, to extrapolate these data to a diagnostic test, we used IHC and digital pathology image analysis for immune infiltrate [22]. Utilising species‐specific IHC for B cells, monocytes/macrophages, and T cells, we observed a higher density of B cells in dogs and higher densities of monocytes/macrophages and T cells in both species in tumour tissues of the CTLA4‐high transcriptomic cohort (Figure 3A–D). Then we utilised ROC analysis to determine how well these variables can discriminate between the two transcriptomic subgroups with clinically relevant sensitivity and specificity. Using the Youden index, we identified optimal cut‐off values for parameters with an area under the curve (AUC) of >0.75. ROC curve analysis demonstrated that monocytes and macrophages could differentiate between transcriptomic subgroups with high sensitivity in both humans and dogs and with high specificity in humans. This indicates that IHC using CD68 and IBA1 antibodies detecting monocyte and macrophages could be used to inform clinical decision‐making based on transcriptomic subtype. In addition, CTLA4 itself (but not MET) was associated with a clinically relevant sensitivity in both species and a clinically relevant specificity in humans (Figure 3E and supplementary material, Figure S3D).

Based on these findings and our RNA‐seq analysis, we recommend that the CTLA4‐high subtype OMMs be characterised as immune ‘hot’, with high levels of T‐cell infiltration and activation, and therefore provision of anti‐CTLA4 may show a favourable response in this subgroup [63]. In contrast, our data suggest the MET‐high tumours are immune ‘cold’ and these patients may have a better response to targeted therapy.

### Tumour microbiota does not correlate with transcriptomic subgroup but may contribute to other pathological features of OMM

**Figure 4:**
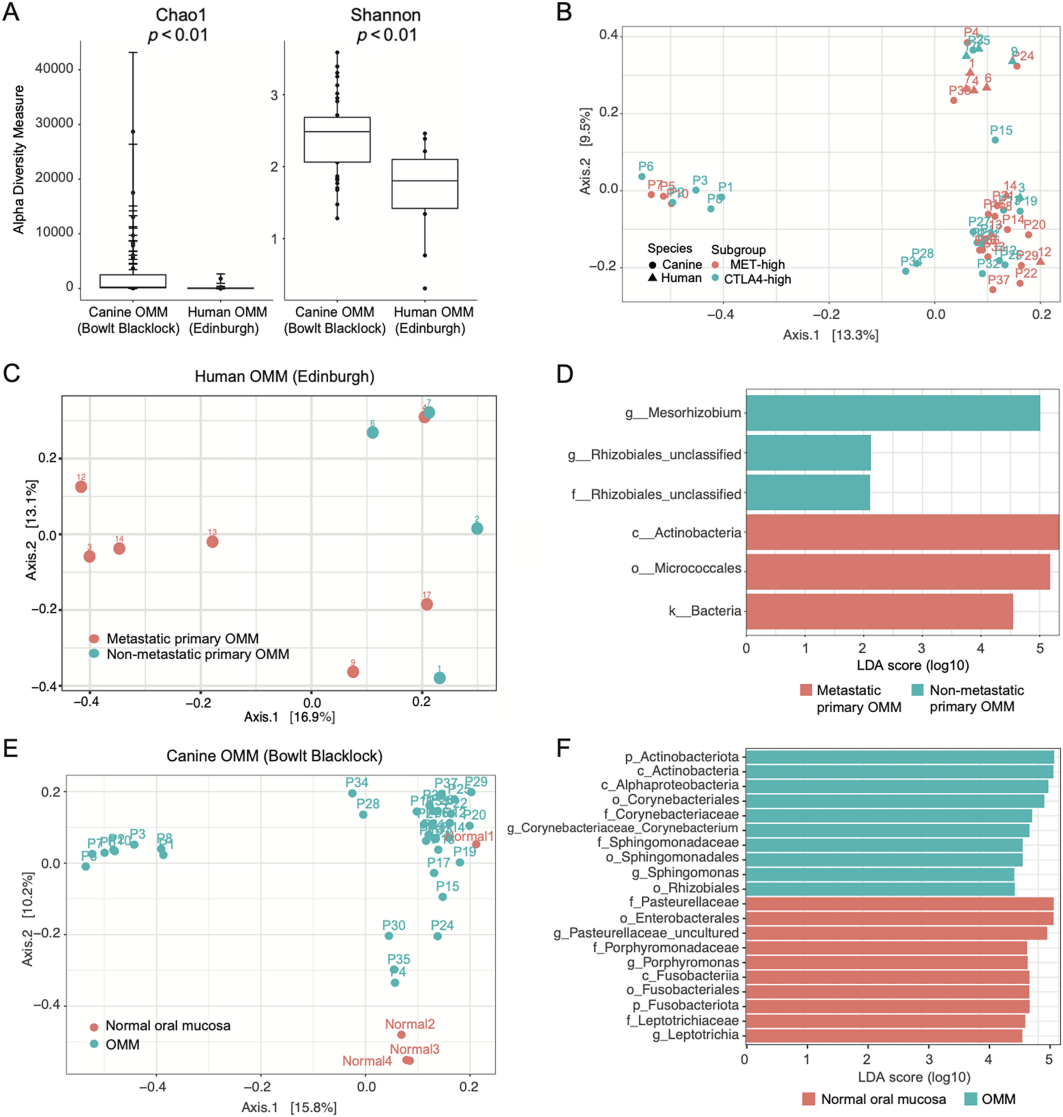
Tumour microbiota does not correlate with transcriptomic subgroup but may contribute to other pathological features of OMM. (A) Box and whisker plot of showing the alpha diversity of human and canine OMM. (B) PCA plot showing beta diversity of human and canine OMM. (C and D) In human OMM, overall community diversity is significantly different between metastatic and non‐metastatic tumours (*p* = 0.03), with three OTU markers identified that are enriched in each group. (E and F) In dogs, the overall community diversity is significantly different between tumour and normal oral tissue (*p* = 0.003), with 17 OTU markers enriched in the OMM primary tumour and 111 OTU markers enriched in the normal oral tissue. The top 10 OTU markers in each enriched group are shown.

The oral cavity hosts the largest and most diverse microbiota after the gut [64]. Given the pivotal role of gastrointestinal tract microbiota in shaping both local and systemic immune system responses [65], we investigated whether the tumour microbiome correlated with our transcriptomic patient stratification, particularly the CTLA4‐high transcriptomic subgroup due to the high immune cell infiltration. To this end, we performed 16S sequencing of DNA extracted from FFPE cores harvested from our human and canine tumour cohorts as well as four normal canine oral tissue samples. Our analysis revealed a significant difference in alpha diversity between species, reflecting the microbiome's diversity within individual samples, with humans exhibiting significantly lower alpha diversity compared to dogs (Figure 4A, *p* < 0.01). Beta diversity, a measure of the similarity or dissimilarity of groups of microbial communities, was significantly different between the two species (*p* < 0.01; Figure 4B and supplementary material, Data S4). Due to the notable differences in alpha and beta diversity observed between species, we conducted separate analyses of the microbiomes associated with human and canine OMM. No significant difference was observed in the microbiome of human or canine OMM when stratified into transcriptomic subtype (Figure 4B). Consequently, the two transcriptomic subgroups of OMM were not attributed to an overall difference in the composition of the oral microbiota. Thus, the immunocyte infiltration that we identified above is likely attributed to inflammation with the primary tumour itself and not bacterial burden.

Although tumour microbiota does not explain the OMM transcriptional subtypes, we did identify two clinically relevant associations that suggest that the tumour microbiome may have a function in OMM disease initiation and progression. First, in human OMM, the overall community diversity was significantly different between metastatic and non‐metastatic tumours (beta diversity *p* = 0.03; Figure 4C), with three OTU markers enriched in each group (Figure 4D). This finding suggests that the microbiota composition may influence the metastatic capacity of OMM [66]. Second, we compared the microbiome profiles of canine OMM with those of normal canine oral tissue and found overall community diversity was significantly different between tumour and normal oral tissue (Figure 4E,F). These data indicate that OMM correlates with alterations in the oral microbiota composition, which warrants further investigation because of the emerging role of the microbiome in cancer development and response to therapy [67, 68, 69].

## Discussion

Our findings provide the conceptional advance that human and canine OMMs are the same disease that can be classified into two subtypes. These two distinct transcriptional subtypes have potential therapeutic implications, and we provide diagnostic tools to distinguish between these two subtypes. Our findings are important for personalised therapeutics for individual patients and for facilitating patient stratification that may inform clinical trial design and outcomes.

The advent of T‐cell‐targeted immunomodulators, such as ipilimumab and nivolumab, and small‐molecule MAPK‐pathway inhibitors has revolutionised cancer care for patients with CM [70, 71, 72]. However, the therapeutic efficacy of immune checkpoint inhibitors for MM remains unclear, and patients with MM are underrepresented in clinical trials [73]. Therefore, outcome data for patients with MM are scarce and mainly based on retrospective studies with limited case numbers. Our two transcriptional subtypes suggest that outcomes and clinical trial design could be improved by patient stratification. The CTLA4‐high subtype exhibits elevated CTLA4 expression and increased immunocyte infiltration, indicative of a ‘hot’ immune tumour type. Patients with CTLA4‐high OMM may benefit from anti‐CTLA4 therapy. In contrast, the MET‐high subtype is characterised by high MET expression and, thus, is potentially responsive to small‐molecule tyrosine kinase inhibitors; the lack of immunocyte infiltration suggests this subtype is a ‘cold’ immune tumour type. Ipilimumab is currently used in human patients, but our review of the literature has not identified clinical data regarding ipilmumab or MET inhibitors as therapeutic approaches for MM. We propose a multicentre retrospective review of treatment efficacy utilising our diagnostic tool to stratify patients into subgroups and examining the impact on outcomes or treatment.

We developed an inexpensive and readily available diagnostic tool which demonstrates that CTLA4, as well as monocytes and macrophages, distinguishes between transcriptomic subgroups with high sensitivity in both humans and dogs. This suggests that IHC utilising antibodies for CTLA4, CD68, and IBA1 offers practical utility for guiding clinical decision‐making based on transcriptomic profiles.

The extent of immune cell infiltration into tumours has important prognostic value in several cancer types [74, 75, 76, 77, 78, 79]. We investigated whether transcriptomic subtype correlated with survival, but, unlike in CM [60, 80, 81, 82], this did not reach significance in either human or canine patients. Transcriptional stratification alone may not predict survival because distinct mechanisms contribute to poor prognosis in each subgroup [83, 84, 85]. However, notably, we did find that increasing age and metastatic disease was associated with poorer survival in both species. The reason for the survival disparity between genders in our human patients with OMM is unknown. While sex disparities in cancer mortality and survival have been reported, particularly for melanoma and cancers of the mouth and nasal cavity [85, 86], the specific impact of sex on survival in OMM patients, as observed in our data, remains to be fully understood. Differential environmental exposures and/or physiological processes may explain these disparities [85].

Interestingly, we found no correlation between microbiome diversity and transcriptomic subgroups, suggesting that tumour‐associated mechanisms, rather than bacterial infection, drive immune infiltration. However, the observed alterations in oral microbiota associated with OMM development in dogs and OMM metastasis in humans should prompt further investigation into the role of microbiota in OMM pathogenesis [87, 88, 89, 90], although we appreciate that caution is advised when applying sequence‐based techniques to the study of microbiota present in low‐biomass environments [91].

In conclusion, we provide valuable insights into the molecular and immune landscapes of OMM and their potential implications for therapeutic interventions. Our findings are potentially enhanced by the homogeneity of our human cohort, and therefore we underscore the importance of future studies prioritising inclusivity by encompassing diverse cohorts [1, 91, 92]. Prospective data collection and tissue sampling efforts will augment the database size and mitigate inherent biases and limitations associated with retrospective data quality and completeness. Moreover, future investigations should explore MM from other anatomical sites to determine the generalisability of our findings across different contexts. The implications of our findings suggest that personalised therapeutic strategies targeting shared molecular pathways could hold promise for both human and canine patients with OMM.

## Author contributions statement

KLBB and EEP conceptualised and designed the study. Data collection was performed by KLBB, JdP, GP, LS, J‐BT, DK, MP, JSM, IB, SZ, SMG, DJS, MST, DM, AM, KP and MC. Data analysis was conducted by KD, YL, DJS, AM, EEP and KLBB. Data interpretation was contributed by LG, MS, MEM, KD, YL, KLBB and EEP. KLBB and EEP conducted the literature search and generated the figures. All authors were involved in writing the paper and had final approval of the submitted and published versions. KLBB and EEP provided supervision and project administration.

## Supporting information


**Figure S1.** Clinical data and Kaplan–Meier survival plots associated with human (Edinburgh) and canine (Bowlt Blacklock) OMM cohorts
**Figure S2.** Two shared transcriptomic subgroups stratify OMM in human and canine patients
**Figure S3.** Violin plots, ROC curves, and Kaplan–Meier survival plots associated with transcriptomic subgroup

**Data S1.** Pathway analysis results associated with two transcriptomic subtypes identified in human and canine OMM

**Data S2.** List of 812 human and canine homologous genes inputted into randomForest machine learning algorithm and genes utilised by the model

**Data S3.** Immunocyte infiltration in human and canine OMM, parsed from annotated gene signature matrix LM22 and 100 permutations of CIBERSORTx web portal

**Data S4.** Beta diversity metrics for microbiome data from human and canine OMM

## Data Availability

De‐identified human clinicopathological and standardised RNA‐seq data were deposited at the European Genome‐Phenome Archive (EGA with accession no. 25513). De‐identified canine clinicopathological, genetic, and standardised RNA‐seq data were deposited at the Sequence Read Archive (SRA with accession no. SUB14547907). De‐identified human and canine standardised 16S sequencing data were deposited at the Sequence Read Archive (SRA with accession no. SUB14547907).

## References

[1] Ossio R , Roldán‐Marín R , Martínez‐Said H , *et al*. Melanoma: a global perspective. Nat Rev Cancer 2017; 17: 393–394. 28450704 10.1038/nrc.2017.43

[2] Elder DE , Bastian BC , Cree IA , *et al*. The 2018 World Health Organization classification of cutaneous, mucosal, and uveal melanoma: detailed analysis of 9 distinct subtypes defined by their evolutionary pathway. Arch Pathol Lab Med 2020; 144: 500–522. 32057276 10.5858/arpa.2019-0561-RA

[3] Newell F , Kong Y , Wilmott JS , *et al*. Whole‐genome landscape of mucosal melanoma reveals diverse drivers and therapeutic targets. Nat Commun 2019; 18: 3163. 10.1038/s41467-019-11107-x PMC6639323 31320640

[4] Nassar KW , Tan AC . The mutational landscape of mucosal melanoma. Semin Cancer Biol 2020; 61: 139–148. 31655118 10.1016/j.semcancer.2019.09.013 PMC7078020

[5] Lian B , Cui CL , Zhou L , *et al*. The natural history and patterns of metastases from mucosal melanoma: an analysis of 706 prospectively‐followed patients. Ann Oncol 2017; 28: 868–873. 28039178 10.1093/annonc/mdw694

[6] Tyrrell H , Payne M . Combatting mucosal melanoma: recent advances and future perspectives. Melanoma Manag 2018; 5: MMT11. 30459941 10.2217/mmt-2018-0003 PMC6240847

[7] Chang AE , Karnell LH , Menck HR . The National Cancer Data Base report on cutaneous and noncutaneous melanoma: a summary of 84,836 cases from the past decade. Cancer 1998; 83: 1664–1678. 9781962 10.1002/(sici)1097-0142(19981015)83:8<1664::aid-cncr23>3.0.co;2-g

[8] Larkin J , Marais R , Porta N , *et al*. Nilotinib in KIT‐driven advanced melanoma: results from the phase II single‐arm NICAM trial. Cell Rep Med 2024; 5: 101435. 38417447 10.1016/j.xcrm.2024.101435 PMC10982988

[9] Seth R , Agarwala SS , Messersmith H , *et al*. Systemic therapy for melanoma: ASCO guideline update. J Clin Oncol 2023; 41: 4794–4820. 37579248 10.1200/JCO.23.01136

[10] Wong K , van der Weyden L , Schott CR , *et al*. Cross‐species genomic landscape comparison of human mucosal melanoma with canine oral and equine melanoma. Nat Commun 2019; 10: 353. 30664638 10.1038/s41467-018-08081-1 PMC6341101

[11] Patton EE , Mueller KL , Adams DJ , *et al*. Melanoma models for the next generation of therapies. Cancer Cell 2021; 39: 610–631. 33545064 10.1016/j.ccell.2021.01.011 PMC8378471

[12] Ablain J , Xu M , Rothschild H , *et al*. Human tumor genomics and zebrafish modeling identify SPRED1 loss as a driver of mucosal melanoma. Science 1979; 2018: 1055–1060. 10.1126/science.aau6509 PMC6475924 30385465

[13] Sun L , Kang X , Ju H , *et al*. A human mucosal melanoma organoid platform for modeling tumor heterogeneity and exploring immunotherapy combination options. Sci Adv 2023; 9: eadg6686. 37889972 10.1126/sciadv.adg6686 PMC10610903

[14] Babu S , Chen J , Robitschek E , *et al*. Specific oncogene activation of the cell of origin in mucosal melanoma. *bioRxiv* 2024; 2024.04.22.590595. [Not peer reviewed].

[15] Prouteau A , Mottier S , Primot A , *et al*. Canine oral melanoma genomic and transcriptomic study defines two molecular subgroups with different therapeutical targets. Cancers (Basel) 2022; 14: 276. 35053440 10.3390/cancers14020276 PMC8774001

[16] Kundra R , Zhang H , Sheridan R , *et al*. OncoTree: a cancer classification system for precision oncology. JCO Clin Cancer Inform 2021; 5: 221–230. 33625877 10.1200/CCI.20.00108 PMC8240791

[17] Bagaev A , Kotlov N , Nomie K , *et al*. Conserved pan‐cancer microenvironment subtypes predict response to immunotherapy. Cancer Cell 2021; 39: 845–865.e7. 34019806 10.1016/j.ccell.2021.04.014

[18] Valenti F , Falcone I , Ungania S , *et al*. Precision medicine and melanoma: multi‐omics approaches to monitoring the immunotherapy response. Int J Mol Sci 2021; 22: 3837. 33917181 10.3390/ijms22083837 PMC8067863

[19] Fountzilas E , Tsimberidou AM , Vo HH , *et al*. Clinical trial design in the era of precision medicine. Genome Med 2022; 14: 101. 36045401 10.1186/s13073-022-01102-1 PMC9428375

[20] Liu Y , Zhu XZ , Xiao Y , *et al*. Subtyping‐based platform guides precision medicine for heavily pretreated metastatic triple‐negative breast cancer: the FUTURE phase II umbrella clinical trial. Cell Res 2023; 33: 389–402. 36973538 10.1038/s41422-023-00795-2 PMC10156707

[21] Duan XP , Qin BD , Jiao XD , *et al*. New clinical trial design in precision medicine: discovery, development and direction. Signal Transduct Target Ther 2024; 9: 57. 38438349 10.1038/s41392-024-01760-0 PMC10912713

[22] Bankhead P , Loughrey MB , Fernández JA , *et al*. QuPath: open source software for digital pathology image analysis. Sci Rep 2017; 7: 16878. 29203879 10.1038/s41598-017-17204-5 PMC5715110

[23] Ewels PA , Peltzer A , Fillinger S , *et al*. The nf‐core framework for community‐curated bioinformatics pipelines. Nat Biotechnol 2020; 38: 276–278. 32055031 10.1038/s41587-020-0439-x

[24] DI Tommaso P , Chatzou M , Floden EW , *et al*. Nextflow enables reproducible computational workflows. Nat Biotechnol 2017; 35: 316–319. 28398311 10.1038/nbt.3820

[25] Quinlan AR , Hall IM . BEDTools: a flexible suite of utilities for comparing genomic features. Bioinformatics 2010; 26: 841–842. 20110278 10.1093/bioinformatics/btq033 PMC2832824

[26] Chen S , Zhou Y , Chen Y , *et al*. fastp: an ultra‐fast all‐in‐one FASTQ preprocessor. Bioinformatics 2018; 34: i884–i890. 30423086 10.1093/bioinformatics/bty560 PMC6129281

[27] Andrews S . FASTQC: a quality control tool for high throughput sequence data, 2010. [Accessed: 13 June 2024]. Available from: https://www.bioinformatics.babraham.ac.uk/projects/fastqc/.

[28] Ewels P , Magnusson M , Lundin S , *et al*. MultiQC: summarize analysis results for multiple tools and samples in a single report. Bioinformatics 2016; 32: 3047–3048. 27312411 10.1093/bioinformatics/btw354 PMC5039924

[29] Patro R , Duggal G , Love MI , *et al*. Salmon provides fast and bias‐aware quantification of transcript expression. Nat Methods 2017; 14: 417–419. 28263959 10.1038/nmeth.4197 PMC5600148

[30] Li H , Handsaker B , Wysoker A , *et al*. The sequence alignment/map format and SAMtools. Bioinformatics 2009; 25: 2078–2079. 19505943 10.1093/bioinformatics/btp352 PMC2723002

[31] Dobin A , Davis CA , Schlesinger F , *et al*. STAR: ultrafast universal RNA‐seq aligner. Bioinformatics 2013; 29: 15–21. 23104886 10.1093/bioinformatics/bts635 PMC3530905

[32] GitHub – FelixKrueger/TrimGalore . A wrapper around Cutadapt and FastQC to consistently apply adapter and quality trimming to FastQ files, with extra functionality for RRBS data. [Accessed May 23, 2024]. Available from: https://github.com/FelixKrueger/TrimGalore.

[33] R: The R Project for Statistical Computing . [Accessed May 23, 2024]. Available from: https://www.r-project.org/.

[34] Love MI , Huber W , Anders S . Moderated estimation of fold change and dispersion for RNA‐seq data with DESeq2. Genome Biol 2014; 15: 550. 25516281 10.1186/s13059-014-0550-8 PMC4302049

[35] Wickham H . ggplot2 (2nd edn). Springer: Cham, 2016.

[36] Neuwirth E . ColorBrewer Palettes [R package RColorBrewer version 1.1‐3]. [Accessed April 2022]. Available from: https://CRAN.R-project.org/package=RColorBrewer.

[37] Morgan M , Obenchain V , Hester JPH . SummarizedExperiment: SummarizedExperiment container. R package version 1.34.0. [Accessed April 2022]. Available from: https://bioconductor.org/packages/SummarizedExperiment.

[38] Frankish A , Diekhans M , Ferreira AM , *et al*. GENCODE reference annotation for the human and mouse genomes. Nucleic Acids Res 2019; 47: D766–D773. 30357393 10.1093/nar/gky955 PMC6323946

[39] Gu Z , Schlesner M , Hübschmann D . cola: an R/Bioconductor package for consensus partitioning through a general framework. Nucleic Acids Res 2021; 49: e15. 33275159 10.1093/nar/gkaa1146 PMC7897501

[40] Zhu A , Ibrahim JG , Love MI . Heavy‐tailed prior distributions for sequence count data: removing the noise and preserving large differences. Bioinformatics 2019; 35: 2084–2092. 30395178 10.1093/bioinformatics/bty895 PMC6581436

[41] Stephens M . False discovery rates: a new deal. Biostatistics 2017; 18: 275–294. 27756721 10.1093/biostatistics/kxw041 PMC5379932

[42] Ignatiadis N , Klaus B , Zaugg JB , *et al*. Data‐driven hypothesis weighting increases detection power in genome‐scale multiple testing. Nat Methods 2016; 13: 577–580. 27240256 10.1038/nmeth.3885 PMC4930141

[43] Durinck S , Moreau Y , Kasprzyk A , *et al*. BioMart and Bioconductor: a powerful link between biological databases and microarray data analysis. Bioinformatics 2005; 21: 3439–3440. 16082012 10.1093/bioinformatics/bti525

[44] Blighe K , Rana SLM . EnhancedVolcano: Publication‐ready volcano plots with enhanced colouring and labeling. R package version 1.14.0. 2022. [Accessed June 2023]. https://github.com/kevinblighe/EnhancedVolcano.

[45] Yu G , Wang LG , Han Y , *et al*. ClusterProfiler: an R package for comparing biological themes among gene clusters. OMICS 2012; 16: 284–287. 22455463 10.1089/omi.2011.0118 PMC3339379

[46] Gene Ontology Consortium , Aleksander SA , Balhoff J , *et al*. The gene ontology knowledgebase in 2023. Genetics 2023; 224: iyad031. 36866529 10.1093/genetics/iyad031 PMC10158837

[47] Kanehisa M , Goto S . KEGG: kyoto encyclopedia of genes and genomes. Nucleic Acids Res 2000; 28: 27–30. 10592173 10.1093/nar/28.1.27 PMC102409

[48] Chen B , Khodadoust MS , Liu CL , *et al*. Profiling tumor infiltrating immune cells with CIBERSORT. Methods Mol Biol 2018; 1711: 243–259. 29344893 10.1007/978-1-4939-7493-1_12 PMC5895181

[49] Denz R , Timmesfeld N . Visualizing the (causal) effect of a continuous variable on a time‐to‐event outcome. Epidemiology 2023; 34: 652–660. 37462467 10.1097/EDE.0000000000001630 PMC10392888

[50] Martin M . Cutadapt removes adapter sequences from high‐throughput sequencing reads. EMBnet J 2011; 17: 10–12.

[51] Schloss PD , Westcott SL , Ryabin T , *et al*. Introducing mothur: open‐source, platform‐independent, community‐supported software for describing and comparing microbial communities. Appl Environ Microbiol 2009; 75: 7537–7541. 19801464 10.1128/AEM.01541-09 PMC2786419

[52] Kozich JJ , Westcott SL , Baxter NT , *et al*. Development of a dual‐index sequencing strategy and curation pipeline for analyzing amplicon sequence data on the miseq illumina sequencing platform. Appl Environ Microbiol 2013; 79: 5112–5120. 23793624 10.1128/AEM.01043-13 PMC3753973

[53] Quast C , Pruesse E , Yilmaz P , *et al*. The SILVA ribosomal RNA gene database project: improved data processing and web‐based tools. Nucleis Acids Res 2013; 41: D590–D596. 10.1093/nar/gks1219 PMC3531112 23193283

[54] McMurdie PJ , Holmes S . Phyloseq: an R package for reproducible interactive analysis and graphics of microbiome census data. PLoS One 2013; 8: e61217. 23630581 10.1371/journal.pone.0061217 PMC3632530

[55] Davis NM , DiM P , Holmes SP , *et al*. Simple statistical identification and removal of contaminant sequences in marker‐gene and metagenomics data. Microbiome 2018; 6: 226. 30558668 10.1186/s40168-018-0605-2 PMC6298009

[56] Oksanen J . Vegan Community Ecology Package [R package vegan version 2.6–4], 2002. [Accessed February 2024]. Available from: https;//github.com/vegandevs/vegan.

[57] Cao Y , Dong Q , Wang D , *et al*. microbiomeMarker: an R/Bioconductor package for microbiome marker identification and visualization. Bioinformatics 2022; 38: 4027–4029. 35771644 10.1093/bioinformatics/btac438

[58] Spitznagel MB , Marchitelli B , Gardner M , *et al*. Euthanasia from the veterinary client's perspective: psychosocial contributors to euthanasia decision making. Vet Clin North Am Small Anim Pract 2020; 50: 591–605. 32115280 10.1016/j.cvsm.2019.12.008

[59] Pegram C , Gray C , Packer RMA , *et al*. Proportion and risk factors for death by euthanasia in dogs in the UK. Sci Rep 2021; 11: 9145. 33947877 10.1038/s41598-021-88342-0 PMC8096845

[60] Thakur R , Laye JP , Lauss M , *et al*. Transcriptomic analysis reveals prognostic molecular signatures of stage I melanoma. Clin Cancer Res 2019; 25: 7424–7435. 31515461 10.1158/1078-0432.CCR-18-3659 PMC7617074

[61] Liaw A , Wiener M . Classification and regression by randomForest. R News 2002; 2: 18–22.

[62] Goding CR , Arnheiter H . MITF—the first 25 years. Genes Dev 2019; 33: 983–1007. 31123060 10.1101/gad.324657.119 PMC6672050

[63] Galon J , Bruni D . Approaches to treat immune hot, altered and cold tumours with combination immunotherapies. Nat Rev Drug Discov 2018; 18: 197–218. 10.1038/s41573-018-0007-y 30610226

[64] Deo PN , Deshmukh R . Oral microbiome: unveiling the fundamentals. J Oral Maxillofac Pathol 2019; 23: 122–128. 10.4103/jomfp.JOMFP_304_18 PMC6503789 31110428

[65] Khan MAW , Ologun G , Arora R , *et al*. Gut microbiome modulates response to cancer immunotherapy. Dig Dis Sci 2020; 65: 885–896. 32067144 10.1007/s10620-020-06111-x PMC7678709

[66] Fu A , Yao B , Dong T , *et al*. Emerging roles of intratumor microbiota in cancer metastasis. Trends Cell Biol 2023; 33: 583–593. 36522234 10.1016/j.tcb.2022.11.007

[67] Liu NN , Ma Q , Ge Y , *et al*. Microbiome dysbiosis in lung cancer: from composition to therapy. NPJ Precis Oncol 2020; 4: 33. 33303906 10.1038/s41698-020-00138-z PMC7730185

[68] Sadrekarimi H , Gardanova ZR , Bakhshesh M , *et al*. Emerging role of human microbiome in cancer development and response to therapy: special focus on intestinal microflora. J Transl Med 2022; 20: 310. 35794566 10.1186/s12967-022-03492-7 PMC9258144

[69] Artemev A , Naik S , Pougno A , *et al*. The association of microbiome dysbiosis with colorectal cancer. Cureus 2022; 14: e22156. 35174040 10.7759/cureus.22156 PMC8840808

[70] Robert C . A decade of immune‐checkpoint inhibitors in cancer therapy. Nat Commun 2020; 11: 3801. 32732879 10.1038/s41467-020-17670-y PMC7393098

[71] Larkin J , Chiarion‐Sileni V , Gonzalez R , *et al*. Five‐year survival with combined nivolumab and ipilimumab in advanced melanoma. N Engl J Med 2019; 381: 1535–1546. 31562797 10.1056/NEJMoa1910836

[72] Robert C , Schachter J , Long GV , *et al*. Pembrolizumab versus ipilimumab in advanced melanoma. N Engl J Med 2015; 372: 2521–2532. 25891173 10.1056/NEJMoa1503093

[73] Wei AZ , Chen LN , Orloff M , *et al*. Proceedings from the Melanoma Research Foundation mucosal melanoma meeting. Pigment Cell Melanoma Res 2023; 36: 542–556. 37804122 10.1111/pcmr.13139

[74] Zeng Y , Zeng Y , Yin H , *et al*. Exploration of the immune cell infiltration‐related gene signature in the prognosis of melanoma. Aging (Albany NY) 2021; 13: 3459–3482. 33428606 10.18632/aging.202279 PMC7906183

[75] Garnelo M , Tan A , Her Z , *et al*. Interaction between tumour‐infiltrating B cells and T cells controls the progression of hepatocellular carcinoma. Gut 2017; 66: 342–351. 26669617 10.1136/gutjnl-2015-310814 PMC5284473

[76] Ali HR , Chlon L , Pharoah PDP , *et al*. Patterns of immune infiltration in breast cancer and their clinical implications: a gene‐expression‐based retrospective study. PLoS Med 2016; 13: e1002194. 27959923 10.1371/journal.pmed.1002194 PMC5154505

[77] Mlecnik B , Bindea G , Angell HK , *et al*. Integrative analyses of colorectal cancer show immunoscore is a stronger predictor of patient survival than microsatellite instability. Immunity 2016; 44: 698–711. 26982367 10.1016/j.immuni.2016.02.025

[78] Hato T , Goyal L , Greten TF , *et al*. Immune checkpoint blockade in hepatocellular carcinoma: current progress and future directions. Hepatology 2014; 60: 1776–1782. 24912948 10.1002/hep.27246 PMC4211962

[79] Rohr‐Udilova N , Klinglmüller F , Schulte‐Hermann R , *et al*. Deviations of the immune cell landscape between healthy liver and hepatocellular carcinoma. Sci Rep 2018; 8: 6220. 29670256 10.1038/s41598-018-24437-5 PMC5906687

[80] Garg M , Couturier DL , Nsengimana J , *et al*. Tumour gene expression signature in primary melanoma predicts long‐term outcomes. Nat Commun 2022; 13: 2841. 35581257 10.1038/s41467-022-30365-w PMC9114317

[81] Cancer Genome Atlas Network . Genomic classification of cutaneous melanoma. Cell 2015; 161: 1681–1696. 26091043 10.1016/j.cell.2015.05.044 PMC4580370

[82] Lauss M , Nsengimana J , Staaf J , *et al*. Consensus of melanoma gene expression subtypes converges on biological entities. J Invest Dermatol 2016; 136: 2502–2505. 27345472 10.1016/j.jid.2016.05.119

[83] Gonzalez H , Hagerling C , Werb Z . Roles of the immune system in cancer: from tumor initiation to metastatic progression. Genes Dev 2018; 32: 1267–1284. 30275043 10.1101/gad.314617.118 PMC6169832

[84] Hartman ML , Czyz M . Pro‐survival role of MITF in melanoma. J Invest Dermatol 2015; 135: 352–358. 25142731 10.1038/jid.2014.319

[85] Cook MB , McGlynn KA , Devesa SS , *et al*. Sex disparities in cancer mortality and survival. Cancer Epidemiol Biomarkers Prev 2011; 20: 1629–1637. 21750167 10.1158/1055-9965.EPI-11-0246 PMC3153584

[86] Smith AJ , Lambert PC , Rutherford MJ . Understanding the impact of sex and stage differences on melanoma cancer patient survival: a SEER‐based study. Br J Cancer 2021; 124: 671–677. 33144697 10.1038/s41416-020-01144-5 PMC7851379

[87] Zhang M , Liu J , Xia Q . Role of gut microbiome in cancer immunotherapy: from predictive biomarker to therapeutic target. Exp Hematol Oncol 2023; 12: 84. 37770953 10.1186/s40164-023-00442-x PMC10537950

[88] Spencer CN , McQuade JL , Gopalakrishnan V , *et al*. Dietary fiber and probiotics influence the gut microbiome and melanoma immunotherapy response. Science 2021; 374: 1632–1640. 34941392 10.1126/science.aaz7015 PMC8970537

[89] Baruch EN , Youngster I , Ben‐Betzalel G , *et al*. Fecal microbiota transplant promotes response in immunotherapy‐refractory melanoma patients. Science 2021; 371: 602–609. 33303685 10.1126/science.abb5920

[90] Salter SJ , Cox MJ , Turek EM , *et al*. Reagent and laboratory contamination can critically impact sequence‐based microbiome analyses. BMC Biol 2014; 12: 87. 25387460 10.1186/s12915-014-0087-z PMC4228153

[91] Molina‐Aguilar C , Robles‐Espinoza CD . Tackling the lack of diversity in cancer research. Dis Model Mech 2023; 16: dmm050275. 37681401 10.1242/dmm.050275 PMC10499025

[92] Qian Y , Johannet P , Sawyers A , *et al*. The ongoing racial disparities in melanoma: an analysis of the surveillance, epidemiology, and end results database (1975–2016). J Am Acad Dermatol 2021; 84: 1585–1593. 32861710 10.1016/j.jaad.2020.08.097 PMC8049091

