# Supplementary material for: Oronasal mucosal melanoma is defined by two transcriptional subtypes in humans and dogs with implications for diagnosis and therapy
Source: J Pathol. 2025 Jan 19;265(3):245–59. doi: 10.1002/path.6377 (PMC11794980; doi:10.1002/path.6377)
Supplement: Supplementary file 1 — Figure S1. Clinical data and Kaplan–Meier survival plots associated with human (Edinburgh) and canine (Bowlt Blacklock) OMM cohorts Figure S2. Two shared transcriptomic subgroups stratify OMM in human and canine patients Figure S3. Violin plots, ROC curves, and Kaplan–Meier survival plots associated with transcriptomic subgroup [file PATH-265-245-s005.zip › path6377-sup-0001-FiguresS1-S3.docx]

**Oronasal mucosal melanoma is defined by two transcriptional subtypes in humans and dogs with implications for diagnosis and therapy**

KLB Blacklock *et al. J Pathol* <https://doi.org/10.1002/path.6377>

**Supplementary Figures S1–S3**

**Supplementary Data S1–S4 (these are provided as separate Excel files)**

**Figure S1. Clinical data and Kaplan Meier survival plots associated with human**

**(Edinburgh) and canine (Bowlt Blacklock) OMM cohorts.** (A) Hazard ratios for survival in human (left) and canine (right) patients with OMM. (B–F) Kaplan–Meier survival plots for canine patients based on (B) tumour ulceration, (C) patient WHO status, (D) tumour exophytic status, (E) tumour melanotic status, and (F) tumour histopathological subtype (F). The y-axis represents survival probability and the x-axis denotes time, with tick marks indicating censored data points. A similar comparison in humans was not possible because of retrospective data availability.

**Figure S2. Two shared transcriptomic subgroups stratify OMM in human and canine**

**patients.** (A) Principal component analysis (PCA) plots for human (Edinburgh)(n=17), canine (Bowlt Blacklock) (n=36), and canine (Prouteau)(n=32) OMM, with cells coloured by transcriptomic subgroup. (B) Venn diagram of 5 models showing 41 genes consistently ranked among the top 100 genes shared by at least 4 models and 17 genes shared by all five models. (C) Box and whisker plots showing MITF expression levels in human and canine OMM according to transcriptomic subgroup.

**Figure S3. Violin plots, ROC curves, and Kaplan–Meier survival plots associated with**

**transcriptomic subgroup.** (A) Violin plots of CIBERSORT “Absolute Score”, representing total immune cell infiltration, in human and canine OMM. (B,C) Violin plots showing the absolute proportion of immune cell types in humans and canine OMM. (D) ROC curves and optimal cut-off parameters for number of MET-positive cells per mm2 in human and canine OMM. (E) Kaplan–Meier plot showing survival stratified by transcriptomic subgroup in human and canine patients with OMM.
