# Supplementary figures and images for: Oronasal mucosal melanoma is defined by two transcriptional subtypes in humans and dogs with implications for diagnosis and therapy
Source: J Pathol. 2025 Jan 19;265(3):245–59. doi: 10.1002/path.6377 (PMC11794980; doi:10.1002/path.6377)

A

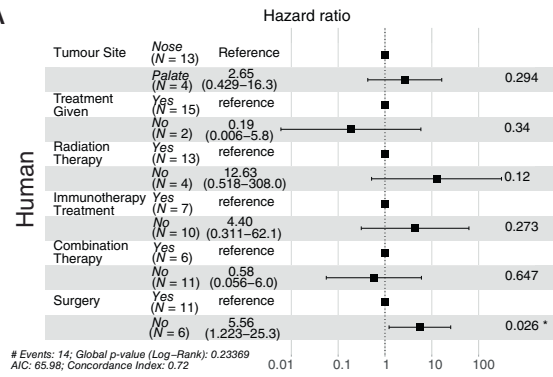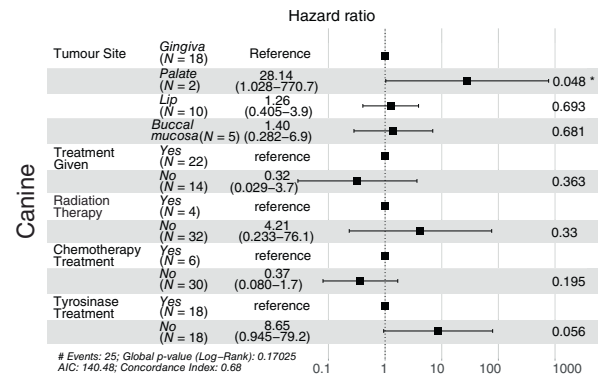

B

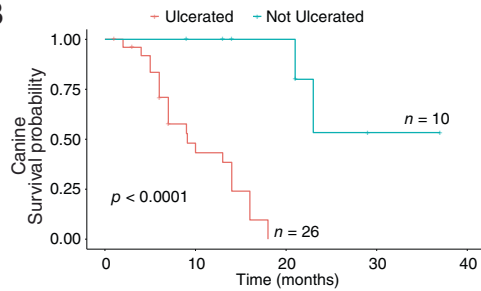

C

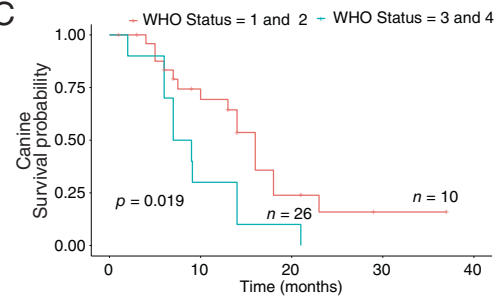

D

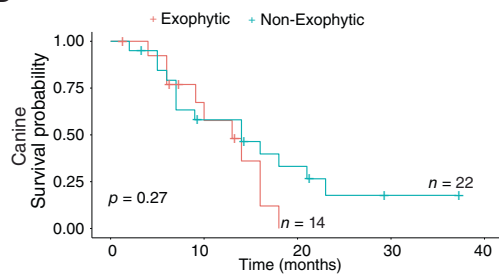

E

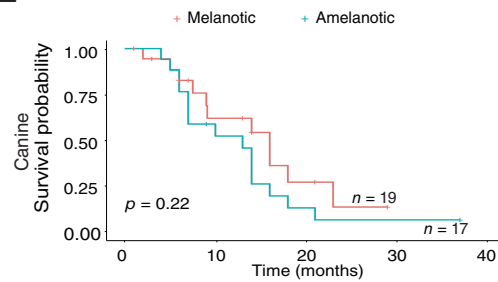

F

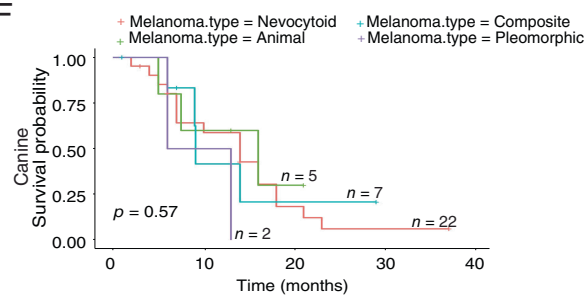

Supplement: Supplementary file 1 — Figure S1. Clinical data and Kaplan–Meier survival plots associated with human (Edinburgh) and canine (Bowlt Blacklock) OMM cohorts Figure S2. Two shared transcriptomic subgroups stratify OMM in human and canine patients Figure S3. Violin plots, ROC curves, and Kaplan–Meier survival plots associated with transcriptomic subgroup [file PATH-265-245-s005.zip › path6377-sup-0002-FigureS1.pdf]

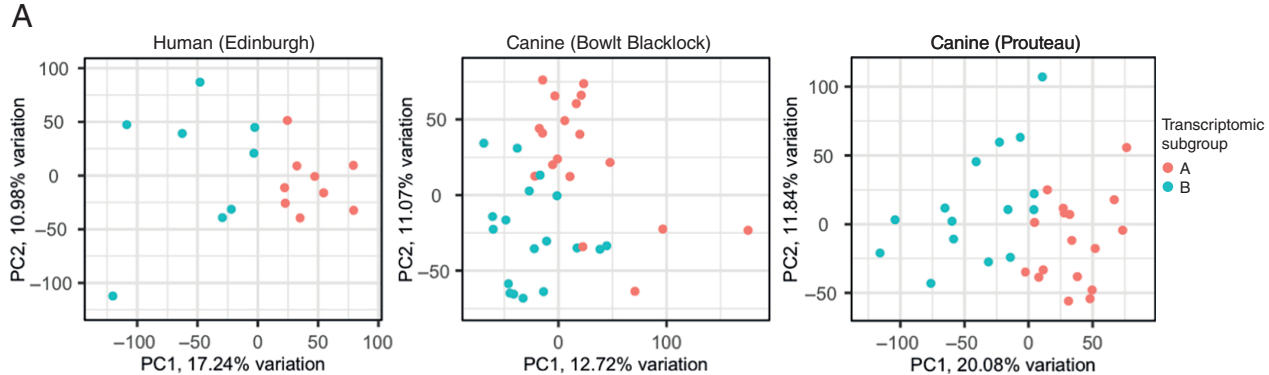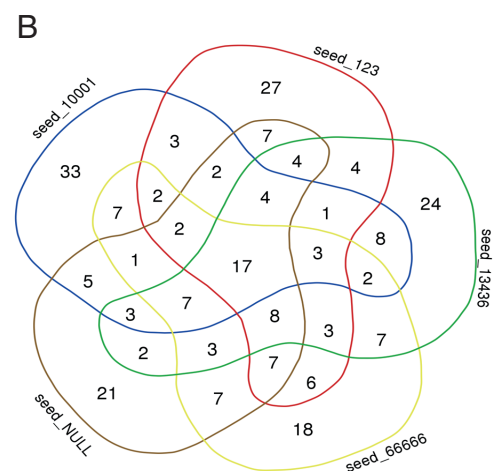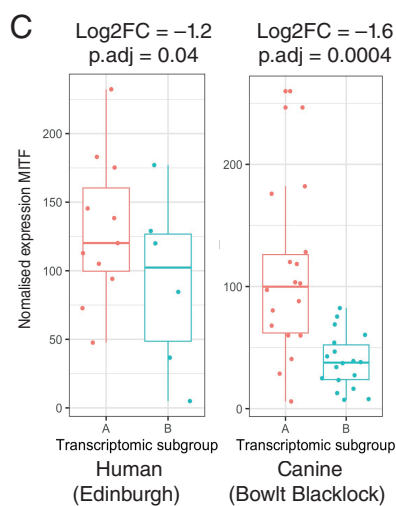

Supplement: Supplementary file 1 — Figure S1. Clinical data and Kaplan–Meier survival plots associated with human (Edinburgh) and canine (Bowlt Blacklock) OMM cohorts Figure S2. Two shared transcriptomic subgroups stratify OMM in human and canine patients Figure S3. Violin plots, ROC curves, and Kaplan–Meier survival plots associated with transcriptomic subgroup [file PATH-265-245-s005.zip › path6377-sup-0003-FigureS2.pdf]

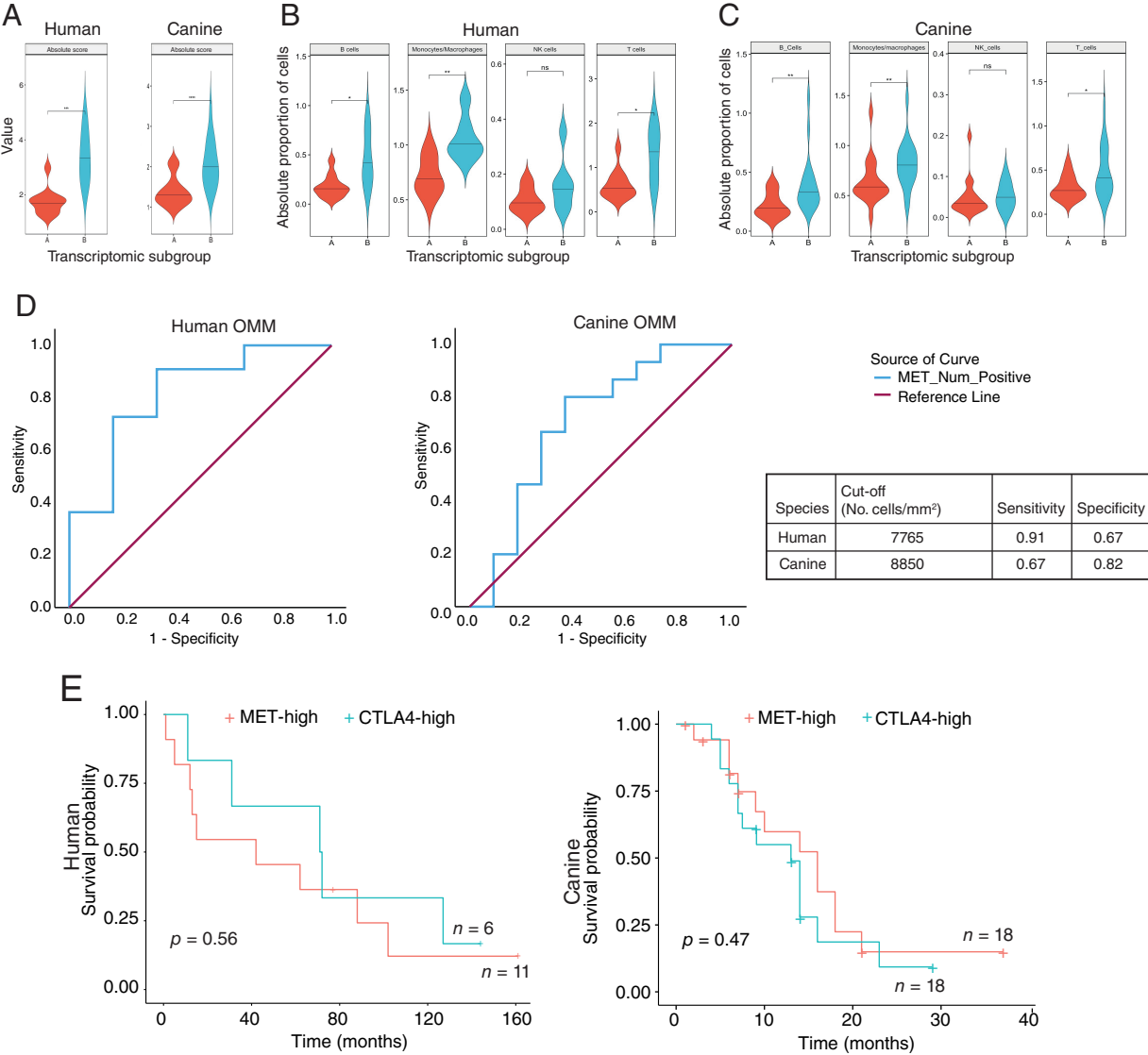

Supplement: Supplementary file 1 — Figure S1. Clinical data and Kaplan–Meier survival plots associated with human (Edinburgh) and canine (Bowlt Blacklock) OMM cohorts Figure S2. Two shared transcriptomic subgroups stratify OMM in human and canine patients Figure S3. Violin plots, ROC curves, and Kaplan–Meier survival plots associated with transcriptomic subgroup [file PATH-265-245-s005.zip › path6377-sup-0004-FigureS3.pdf]
